# Downsized: gray whales using an alternative foraging ground have smaller morphology

**DOI:** 10.1098/rsbl.2023.0043

**Published:** 2023-08-09

**Authors:** K. C. Bierlich, A. Kane, L. Hildebrand, C. N. Bird, A. Fernandez Ajo, J. D. Stewart, J. Hewitt, I. Hildebrand, J. Sumich, L. G. Torres

**Affiliations:** ^1^ Geospatial Ecology of Marine Megafauna Lab, Oregon State University, Corvallis, Oregon, USA; ^2^ Ocean Ecology Lab, Marine Mammal Institute, Department of Fisheries, Wildlife and Conservation Sciences, Oregon State University, Corvallis, Oregon, USA; ^3^ Department of Statistical Science, Duke University, Durham, NC, USA; ^4^ Marine Mammal Institute, Department of Fisheries, Wildlife and Conservation Sciences, Oregon State University, Corvallis, Oregon, USA

**Keywords:** morphology, ecological niche, photogrammetry, Bayesian, gray whales, von Bertalanffy growth curve

## Abstract

Describing individual morphology and growth is key for identifying ecological niches and monitoring the health and fitness of populations. Eastern North Pacific ((ENP), approximately 16 650 individuals) gray whales primarily feed in the Arctic/sub-Arctic regions, while a small subgroup called the Pacific Coast Feeding Group (PCFG, approximately 212 individuals) instead feeds between northern California, USA and British Columbia, Canada. Evidence suggests PCFG whales have lower body condition than ENP whales. Here we investigate morphological differences (length, skull, and fluke span) and compare length-at-age growth curves between ENP and PCFG whales. We use ENP gray whale length-at-age data comprised of strandings, whaling, and aerial photogrammetry (1926–1997) for comparison to data from PCFG whales collected through non-invasive techniques (2016–2022) to estimate age (photo identification) and length (drone-based photogrammetry). We use Bayesian methods to incorporate uncertainty associated with morphological measurements (manual and photogrammetric) and age estimates. We find that while PCFG and ENP whales have similar growth rates, PCFG whales reach smaller asymptotic lengths. Additionally, PCFG whales have relatively smaller skulls and flukes than ENP whales. These findings represent a striking example of morphological adaptation that may facilitate PCFG whales accessing a foraging niche distinct from the Arctic foraging grounds of the broader ENP population.

## 1. Introduction

Morphological traits (i.e. body size and proportions) are fundamental mechanisms for adaptive radiation and speciation [1,2]. Individual growth rates are susceptible to environmental variability and predation risk, which can influence life-history traits, such as size or age at maturity, reproductive potential, and resilience to resource limitations [3–6]. Thus, modelling growth processes is key for monitoring the health of populations and their vulnerability to environmental and anthropogenic stressors [7–11].

Obtaining size-at-age data for cetaceans is logistically challenging and most current knowledge on growth and morphology relies on specimens collected from whaling, bycatch and strandings [12–17]. However, these data records lack repeated measurements of individuals over time, which can bias population-level length–age curves [4,18]. Non-invasive approaches for obtaining size and age information of cetaceans are therefore crucial to better identify differences between populations, ecological drivers of speciation, and appropriate management strategies.

Photo-identification and re-sighting histories of individuals over time provide estimates of age from date of first sighting [15,19–22]. Aerial, underwater and laser photogrammetry provide opportunities for non-invasive length measurements of live cetaceans [23–28]. In particular, the recent proliferation of drones (unoccupied aircraft systems, UAS) provides an accessible alternative for aerial photogrammetry compared to crewed aircraft [29–31]. Together, photo-identification history and photogrammetry-based length measurements of individuals enables a non-invasive approach for obtaining age and length measurements to generate growth models for cetacean populations [25,32,33]. Using these paired techniques, several studies have documented decreases in body size across generations, attributed to nutritional stress, entanglements and/or vessel strikes [33–35]. Such decreases in body size are associated with reductions in reproductive success and survival [6,36,37].

Eastern North Pacific (ENP) gray whales (*Eschrichtius robustus*) (approx. 16 650 individuals, [38,39]) migrate between their wintering grounds along Baja California, Mexico to their summer foraging grounds in the Bering, Chukchi, and Beaufort Seas (hereafter referred to as ‘Arctic’). A subgroup, called the Pacific Coast Feeding Group (PCFG, approximately 212 individuals, [40]), instead truncates their migration and forages along the coastline between Northern California, USA and British Columbia, Canada (41̊N–52̊N) between 1 June and 30 November [41]. While PCFG whales show high site fidelity to this range [42], it is unknown why PCFG whales use this foraging ground. Despite evidence that prey in the PCFG range is of equal or higher caloric value than prey in the Arctic range [43], recent evidence suggests PCFG whales generally have poorer body condition than ENP whales, likely due to regional differences in prey availability [44]. Long-term photo-identification histories extending back to 1972, which helped first identify this subgroup [22,45], provide data to estimate the age of individual PCFG whales.

Here we use ages from photo-identification history and UAS-based length measurements of PCFG whales, and data collected via whaling, strandings and aerial photogrammetry studies on ENP whales [16,17], to compare morphology and growth of gray whales targeting these distinct foraging regions. We use a novel Bayesian approach to incorporate uncertainty associated with morphological measurements and age estimates. We identify significant differences in growth and morphology between ENP and PCFG, with implications for ecological–evolutionary dynamics, population designations and management.

## 2. Methods

We collected UAS videos and extracted snapshots of PCFG whales off Oregon, USA 2016–2022 (*n* = 70 individuals) [45] (electronic supplementary material, table S1 includes UAS, camera and altimeter specifications). All UAS are susceptible to photogrammetric uncertainty associated with the altimeter, camera, focal length, and pixel measurement, so we applied Bayesian methods to incorporate this uncertainty to estimate a posterior distribution for each morphological measurement [46,47]. ENP gray whale length-at-age has been studied previously using a large dataset (*n* = 730) comprised of length data from strandings, whaling, and aerial photogrammetry (1926–1997) [16]. For our analysis, we included post-weaning whales from this dataset (*n* = 419) (Phase 2 growth, age greater than 0.8 years, [16]). While unaccounted for in their analysis [16], a subset (*n* = 151) of these data also contains skull and fluke measurements collected via whaling 1959–1969 off California [17]. Unlike measurements from commercial whaling, which are susceptible to bias from whalers targeting larger whales and/or falsifying records under the legal limit [48], the objective of this ENP whaling operation was to collect a representative sample of the population across demographic unit for ecological analysis [17]. However, body length measurements from this whaling data may have ‘stretched’ up to 7% while towing the carcass, which was unaccounted for in their growth model [16]. We use Bayesian methods to account for ‘stretching’ bias in this subset by using body length adjusted measurements.

We use von Bertalanffy–Putter growth curves [16,49,50] to model ENP and PCFG expected length (*L*) at age (*t*):

$$L_{t}=A(1-e^{-K(t-t_{0})}),$$

where *A* is asymptotic length, *K* is growth rate and *t*_0_ is theoretical age when size is 0. Differences between ENP and PCFG growth are estimated using a joint Bayesian modelling framework to propagate uncertainty. We use uninformative priors for *A*, *K* and *t*_0_ for both ENP and PCFG (electronic supplementary material, table S2 includes dataset descriptions).

**Figure 1.**
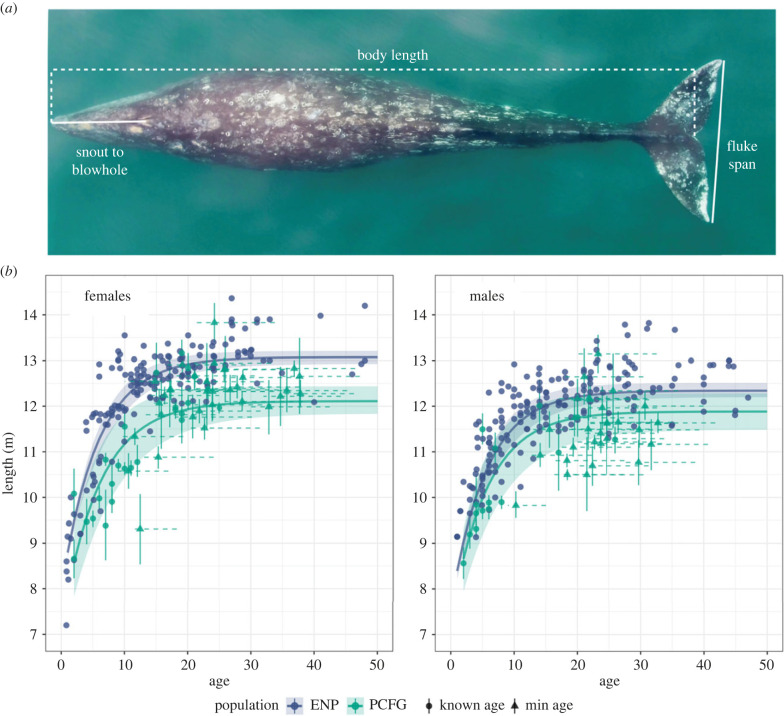
(*a*) Morphological measurements for known Eastern North Pacific (ENP) and Pacific Coast Feeding Group (PCFG) gray whales. (*b*) von Bertalanffy–Putter growth curves for length-at-age comparing male and female ENP and PCFG gray whales (shading represents 95% highest posterior density intervals). Points represent mean length and median age. Vertical bars represent photogrammetric uncertainty. Dashed horizontal lines represent uncertainty in age estimates.

Morphological measurements from both study regions include body length (snout-to-fluke notch), skull length (snout-to-blowhole), and fluke span (tip-to-tip) (figure 1*a*) and were measured manually [17] or photogrammetrically using MorphoMetriX [51] and CollatriX [52]. We estimated PCFG whales' age using date of first sighting from photo-identification history to assign ‘minimum age’, or ‘known age’ if individual was first seen as a calf. We excluded individuals with minimum age ≤ 8, the mean age at sexual maturity [17], to ensure minimum age estimates were from mature individuals. ENP age was determined from earplug growth layers (one per year) [53]. Sex was determined via examination [17], observation (mother with calf), or genetically from faecal analysis [54].

Rather than assume that whales measured in the PCFG range were members of the PCFG, we allowed the model to probabilistically assign group membership (ENP or PCFG) using a mixture approach. We simultaneously estimated mean skull length and fluke span for ENP and PCFG whales in the growth model to identify potential morphological differences between foraging regions and help assign whales to a group. See electronic supplementary material for details of modelling approach.

## 3. Results

**Figure 2.**
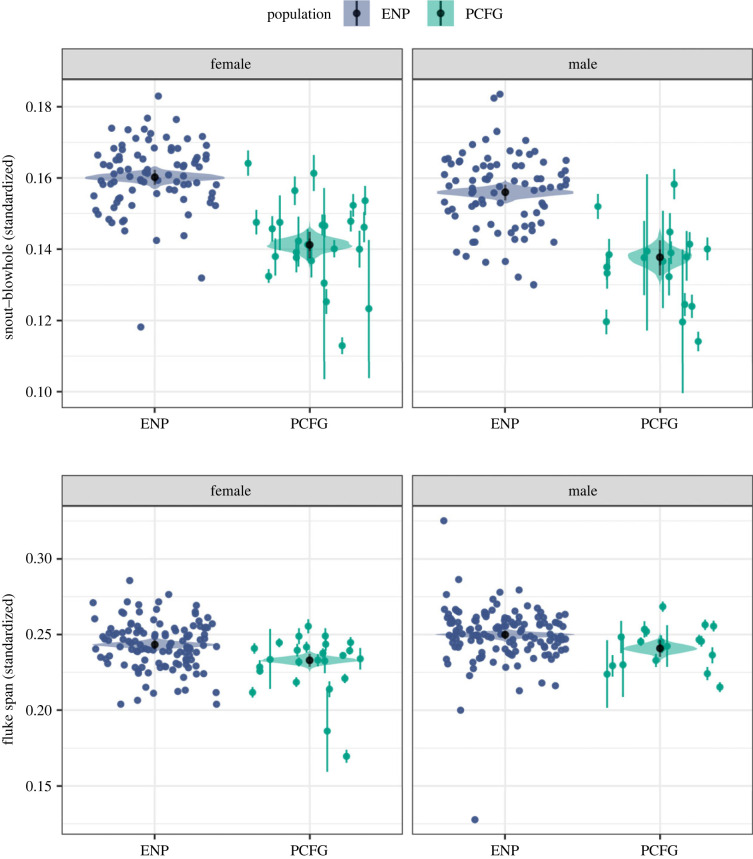
Eye plots representing the posterior distributions for the estimated mean snout–blowhole and fluke span measurements (standardized by length) for known male and female Eastern North Pacific (ENP) and Pacific Coast Feeding Group (PCFG) gray whales; black dot and bars represent median and 95% highest posterior density intervals, respectively. Points represent measurements with vertical bars representing the photogrammetric uncertainty.

We found that PCFG gray whales have significantly shorter body lengths, skulls and flukes than historical ENP whales (table 1, figures 1 and 2, electronic supplementary material, figures S1–S4). Throughout the results, we report the median parameter estimates with 95% highest posterior density intervals in parentheses. While males and females in both populations have similar growth rates, PCFG females reached an asymptotic length 0.96 m (0.62, 1.28) shorter than ENP females, and PCFG males reached an asymptotic length 0.46 m (0.03, 0.87) shorter than ENP males (table 1, figure 1, electronic supplementary material, figures S1 and S2). Within ENP, females reach asymptotic lengths 0.73 m (0.53, 0.93) larger than males. Whereas PCFG females were not significantly larger than PCFG males (table 1, figure 1, electronic supplementary material, figures, S1 and S2).

**Table 1.** Median parameter estimates (95% highest posterior density interval) for known male and female Eastern North Pacific (ENP) and Pacific Coast Feeding Group (PCFG) gray whales.

population, sex	growth			skull length (snout to blowhole as proportion of body length)		fluke span (tip to tip, as proportion of body length)	
	*n* (*n*_r_)	*K*	*A*	*n* (*n*_r_)		*n* (*n*_r_)	
ENP females	191 (0)	0.174 (0.154, 0.195)	13.08 (12.93, 13.21)	86 (0)	0.160 (0.158, 0.162)	118 (0)	0.243 (0.240, 0.246)
PCFG females	38 (14)	0.166 (0.131, 0.207)	12.11 (11.84, 12.44)	25 (11)	0.141 (0.138, 0.145)	25 (11)	0.233 (0.228, 0.237)
ENP males	228 (0)	0.178 (0.154, 0.201)	12.34 (12.20, 12.51)	84 (0)	0.156 (0.154, 0.158)	133 (0)	0.250 (0.247, 0.252)
PCFG males	32 (6)	0.176 (0.142, 0.215)	11.88 (11.49, 12.29)	19 (6)	0.141 (0.133, 0.143)	19 (6)	0.241 (0.235, 0.246)

*K* = growth rate; *A* = asymptotic length (in metres); *n* = number of samples; *n*_r_ = number of individuals with repeated measurements (see electronic supplementary material, table S2 and S3 for more details on sample size and repeated measurements); *t*_0_ = theoretical age when size is 0 and is the same for each population and sex, −5.42 (−6.25, −4.63). Note, ENP whales with an unknown sex (*n* = 55) were probabilistically assigned by the model based on length and age, *n* = 17 females, *n* = 38 males.

PCFG females and males have skulls (as percent of length) 1.90% (1.49%, 2.32%) and 1.84% (1.28%, 2.34%) smaller than ENP females and males, respectively (figure 2, electronic supplementary material, figure S3). We detected a small but significant difference in skull size between ENP females and males, 0.42% (0.12%, 0.71%), and no significant difference between PCFG females and males, 0.34% (−0.22%, 0.95%) (electronic supplementary material, figure S3). PCFG females and males have slightly smaller flukes than ENP females and males, a difference of 1.03% (0.53%, 1.56%) and 0.90% (0.21%, 1.49%) respectively (figure 2, electronic supplementary material, figure S4). Interestingly, males have slightly larger flukes than females in both populations, 0.66% (0.23%, 1.08%) for ENP and 0.78% (0.08%, 1.47%) for PCFG (figure 2, electronic supplementary material, figure S4). Consequently, 69 of 70 PCFG individuals had a >80% probability of being assigned to the PCFG, while one whale had a 60–80% probability (electronic supplementary material, figures S5 and S6).

## 4. Discussion

We present evidence that PCFG gray whales reach shorter body lengths and have shorter skulls and flukes than ENP whales. ENP whales have not experienced significant changes in body length since the 1970s [16] and the mean body length of ENP lactating females imaged photogrammetrically 1994–1998 (12.4 m, range: 11.2–13.9, *n* = 98; [55]) and 2017–2019 (12.4 m, range: 10.73–13.7, *n* = 260; [56]) are similar to our results (12.66 m, range: 10.54–14.2, *n* = 118; [17]). While historical measurements of PCFG whales are rare, three of four lactating PCFG females observed in aerial surveys 1978–1980 were less than 10.5 m [57], well below the smallest reported lactating ENP female [17,55,56]. Therefore, we posit that ENP body length has not changed over the past half century, while PCFG have remained smaller.

Pressing questions arise, including: when did the PCFG form? While results from microsatellite allele frequencies show no genetic difference between ENP and PCFG, evidence supports differences in mitochondrial DNA haplotype frequencies, suggesting that matrilineal fidelity to this foraging ground contributes to recruitment into the PCFG [58–60]. Indeed, most calves first sighted in the PCFG range are re-sighted as juveniles or adults, further supporting matrilineal fidelity as a significant role in creating population structure [42,61]. The level of external recruitment into the PCFG is unknown, but our results suggest it may be low given that none of the PCFG whales had morphological traits similar to ENP (electronic supplementary material, figures S5 and S6). While PCFG whales were first documented within our Oregon Coast study site in the 1970s (electronic supplementary material, figure S7), presumed PCFG gray whales have been reported foraging June–September off the coasts of Northern California to British Columbia since the 1920s [17,62–65]. The Makah Tribe (Washington Coast, USA) traditionally hunted gray whales from October to June, but this is generally outside summer residency of PCFG whales [41]. It is possible that colonization/recolonization has occurred several times in the PCFG range due to commercial whaling or other climatic events (i.e. Little Ice Age) [58–60].

Why are PCFG whales smaller? According to Bergmann's Rule, larger individuals are typically found at higher latitudes [66,67], which could explain why ENP whales are longer and fatter given their longer migration to colder Arctic feeding grounds [68]. The genetic similarity, but different asymptotic lengths between ENP and PCFG may suggest differences in phenotypic plasticity [4,5]. Globally, many terrestrial and aquatic species, including cetaceans, have experienced reductions in body size within the past century, attributed to changes in habitat, food availability, and/or anthropogenic disturbances [5,7,35,69–72]. While both populations are vulnerable to perturbations in food availability [54,56,73], regional prey quality and availability impacts ENP and PCFG gray whales differently, and PCFG have lower body condition than ENP whales [44]. Gray whales face several anthropogenic threats (entanglements, vessel strikes, ocean noise, pollutants [74–76]), and these stressors may be more prevalent in the PCFG range compared to the Arctic foraging grounds given proximity to major population centres, which could plausibly contribute to increased stress [74] and restricted growth of PCFG whales.

The smaller morphology of PCFG may also be related to foraging tactics employed on different prey and habitat types. Differences in skull and fluke morphology are associated with differences in habitat, feeding strategies, prey types, and hydrodynamics among baleen whales [77–81]. Gray whales are considered ‘slow manoeuvrers’ compared to other baleen whales, enabling them to employ flexible foraging tactics on various prey types [77,82–84]. ENP whales feeding in the Arctic generally forage on benthic amphipods, while PCFG whales switch between benthic, epibenthic and planktonic prey, but often target epibenthic mysids [43,83–87]. The feeding tactic employed by gray whales varies based on prey type [84,88]. For instance, gray whales feeding on mysid swarms, which are patchy and ephemeral, have shorter dives, fewer surfacings with less respirations, and swim faster than gray whales feeding on benthic amphipods [83–85,87,88]. Within the PCFG range, gray whales often forage in rocky kelp beds close to shore in water depths (approx. 10 m) that are on average four times shallower than whales feeding in the Chukchi Sea [43]. Thus, the PCFG range may serve as an ecological opportunity, where an alternative habitat with high-caloric prey [43] provides new resource availability, but one that favours a smaller and less buoyant [89–91] morphology for capturing prey in shallower, more complex habitat [92]. This ecological opportunity may also favour smaller whales that are less competitive on the Arctic feeding grounds.

Key questions remain regarding potential consequences of smaller sized PCFG whales, such as reduced resilience to environmental and anthropogenic stressors, or fecundity and population fitness [11,37]. In combination with differences between PCFG and ENP body condition [44], our results encourage re-evaluating population management designations of gray whales in the Eastern North Pacific to consider the PCFG as a separate management unit that likely requires different management strategies than ENP gray whales.

## Data Availability

Data and code are hosted in the figshare open access repository: https://doi.org/10.6084/m9.figshare.22584451 [93]. The data are provided in the electronic supplementary material [94].
